# Vulnerability profiles and prevalence of HIV and other sexually transmitted infections among adolescent girls and young women in Ethiopia: A latent class analysis

**DOI:** 10.1371/journal.pone.0232598

**Published:** 2020-05-14

**Authors:** Carly A. Comins, Katherine B. Rucinski, Stefan Baral, Samuele A. Abebe, Andargachew Mulu, Sheree R. Schwartz

**Affiliations:** 1 Department of Epidemiology, Johns Hopkins Bloomberg School of Public Health, Baltimore, MD, United States of America; 2 Armauer Hansen Research Institute (AHRI), Addis Ababa, Ethiopia; University of Westminster, UNITED KINGDOM

## Abstract

**Background:**

Adolescent girls and young women (AGYW) aged 15–24 years have among the highest risk for HIV and other sexually transmitted infections (STI) across sub-Saharan Africa. A latent class analysis (LCA) was conducted to identify intersecting social- and structural-level determinants of HIV/STI acquisition among AGYW in Ethiopia.

**Methods:**

AGYW were recruited from venues using time-location sampling, completing an interviewer-administered behavioral survey and biological testing for HIV, syphilis, and chlamydia. LCA was used to identify distinct groups, defined by social- and structural-level determinants of HIV/STI risk, among AGYW. Prevalence ratios (PR) and 95% confidence intervals (CI) compared differences in HIV/STI prevalence by group.

**Results:**

A total of 1,501 AGYW were enrolled across Addis Ababa (March–May 2018) and Gambella (June–July 2019). We identified three patterns of vulnerability defined by schooling status, migration history, food insecurity, orphan status, social support, and employment. We labeled these groups as “highly vulnerable” (representing ~21% of the population), “stable, out-of-school, migrated” (~42%), and “stable, in-school, never migrated” (~37%). STI prevalence was nearly two-fold higher among AGYW in the “highly vulnerable” group compared to AGYW in the “stable, in-school, never migrated” group (PR 1.93; 95% CI 1.33, 2.80).

**Conclusions:**

Characterizing patterns of vulnerability among AGYW that reflect higher-level social and structural factors can help facilitate early identification of AGYW at the highest risk of HIV/STI acquisition, thus differentiating groups of AGYW who may most benefit from targeted HIV prevention interventions during adolescence and early adulthood.

## Introduction

In countries across the continent of Africa, nearly 60% of people are under the age of 25 and it is the only continent in the world where the population of youth is growing [1, 2]. Approximately two-thirds of global HIV incident infections in 2017 occurred in countries across sub-Saharan Africa with the majority of those being in Southern and Eastern Africa [3]. Notably, a third of all new HIV infections in countries across sub-Saharan Africa occurred among youth aged 15–24 [3].

In Ethiopia, a country of more than 106 million, more than 610,000 people are living with HIV [3, 4] and HIV prevalence is estimated as 2.9% among those 15–49 years of age [5]. However, there is substantial heterogeneity across geographic areas and populations [6], and the cities of Gambella and Addis Ababa have the highest HIV prevalence nationally (4.8% and 3.4%, respectively). Similar to other countries in the region, women of reproductive age are twice as likely to be living with HIV [5] than men aged 15–49. Moreover, adolescent girls and young women (AGYW) aged 15–24 are up to three times as likely to be living with HIV compared to their male counterparts [5, 7]. Ethiopia is also rapidly urbanizing, with the urban population projected to triple by the year 2037 [8]. Given that AGYW are thought to comprise more than half of all urban migrants in Ethiopia [9], there is an urgent need to prioritize and identify groups of AGYW who are most vulnerable to HIV and other STIs.

HIV risk among AGYW is explained through a confluence of factors, including biological, individual, social, and structural factors embedded within the local HIV epidemic [10, 11]. While HIV risks and vulnerabilities among AGYW are not evenly distributed, factors influencing HIV risk include early sexual debut, age-disparate or intergenerational sex, violence and sexual abuse, and transactional sex including the exchange of sex for money or goods [12–19]. Yet these more proximal HIV and STI risks likely reflect a complex interplay of both social and structural factors which inform behavioral context, including economics, education, gender norms, and social support [20–26].

Latent class analysis (LCA) is a statistical method used to evaluate multiple risk factors simultaneously to uncover classes, or groups of individuals, with distinct patterns of characteristics [27, 28]. Utilizing a person-centered framework [27, 29], LCA methods have been used to examine sexual partnership types [30, 31], sexual risk behaviors [32–35], and latent constructs of stigma [36]. Here, we use LCA methods to characterize patterns of structural and social vulnerability, and their association with STI and HIV prevalence, among AGYW in Ethiopia.

## Materials and methods

### Study setting, population, and data collection procedures

**Fig 1:**
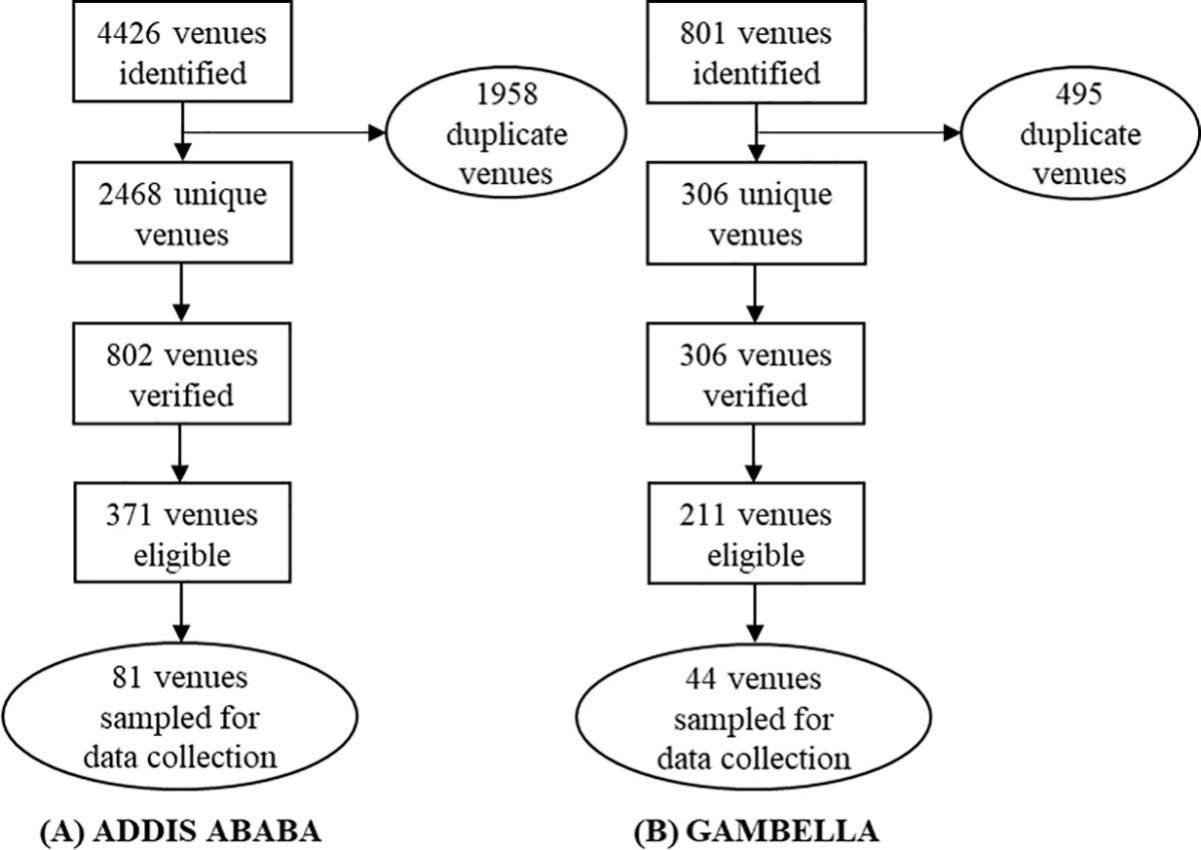
Flowchart of venue mapping activities in (A) Addis Ababa and (B) Gambella.

Data were collected from venues identified through venue mapping and time location sampling in three sub-cities of Addis Ababa (Kolfe Keranio, Addis Ketema, and Akaki Kality) and in Gambella Town [37]. Study procedures have been previously described [38]. Briefly, community-based key informant discussions identified venues (e.g., bars/restaurants, broker houses, brothels, etc.) or locations where vulnerable, predominately out-of-school AGYW hang out, work, or congregate to meet friends or boyfriends. Venues were then validated for inclusion in the sampling frame. Venues with high levels of AGYW congregation (eight or more AGYW onsite each hour) were eligible to be sampled for data collection [38, 39]. Venue enumeration and sampling flow are summarized in Fig 1. Venues were then randomly selected from all eligible venues for final inclusion in the study (81 and 44 randomly selected venues in Addis Ababa and Gambella, respectively) based on the total sample size for each city and enrollment targets per venue.

During the selected day and time block at each venue, AGYW were systematically approached by the study team to explain the nature of the study and if interested, screen for eligibility; study teams carried letters of approval from the Ministry of Health. AGYW aged 15–24 years old were eligible for enrollment. For AGYW uninterested in learning more about the study or eligible but refusing to participate, no information was collected for those individuals. Participants completed an interviewer-administered face-to-face structured survey using tablets (REDCap^TM,^ Nashville, TN) and provided 5 mL of whole blood for HIV, syphilis, and chlamydia testing. The survey was conducted in a private space, lasting approximately 30–45 minutes and captured demographics, marriage and sexual history, reproductive health, prior HIV and STI-testing/treatment histories, history of physical and sexual violence, and an assessment of social support and mental health. Questionnaires were offered in Amharic, English, and Nuer. Consistent with national guidelines, pre- and post-test counseling were included with HIV, syphilis, and chlamydia testing and participants testing positive were referred to a local health facility for treatment.

Ethical review was obtained from the Armauer Hansen Research Institute ethical review board, the Ethiopian National Research Ethics Committee, and the Institutional Review Board at Johns Hopkins Bloomberg School of Public Health. Written informed consent was granted from all AGYW prior to participation in the study, including those AGYW aged 15–17 with emancipated minor status as per rules and regulations established by Ethiopian Research Guidelines [40]; parental consent for those under 18 was thus waived by the IRB. In Addis Ababa and Gambella, AGYW received 300 and 200 Ethiopian Birr for their participation, respectively (USD ~10 and USD~7), which is consistent with local research reimbursement standards.

### Measures

AGYW vulnerability was assessed using items that measured higher order social and structural factors known to facilitate STI/HIV acquisition and transmission among AGYW. Potential factors for inclusion were informed by a modified socio ecological framework and identified through a literature review [22, 24, 25, 41–43]. Measures included in the analysis comprised schooling status (in school/out-of-school), migration history (migrated/never migrated), food insecurity in the last four weeks (none/yes, rarely/yes, sometimes/often), orphan status (both parents living/one parent living/no parents living), social support based on the 12-item version of the social support survey scale (MOS-SSS) (tertiles: low/medium/high), and employment status (unemployed/informally employed/employed in public or private sector).

### Statistical analysis

Characteristics for AGYW were described overall and by sexually active status (Stata version 15.0; College Station, Texas) [44, 45] and we used LCA to identify and describe underlying constructs of social and structural HIV-associated risk factors. Models were fit using PROC LCA, an add-on package to SAS statistical software (SAS, version 9.4, Cary, NC). All models accounted for clustering by city (Addis Ababa/Gambella) and included robust standard errors. We considered models with up to six classes, beginning with a two-class model and increasing the number of classes until the Bayesian Information Criterion (BIC) stopped decreasing [46]. Model identification was assessed by generating 100 random starting values [46]; in cases where nearly 80% of the seeds converged to the same solution, the model was considered identified and suitable for further analysis [28]. For all models, we examined the conditioned probabilities and the latent class proportions for the included variables. Models considered for further analysis were those where the mean and median posterior probabilities of class membership were >0.70 [47]. In selecting the final model, interpretability was also considered; that is, whether the latent classes were logical given the study population, were themselves distinct, and could readily be labeled.

Once an optimal model was selected, each AGYW was then “assigned” to a latent class (group) using a maximum probability assignment rule (i.e. assigned to the latent class group for which her posterior probability of group membership was highest) [28]. We used the latent class proportions for the social and structural HIV-associated risk factors included in the model to interpret and name the groups. More proximal individual and behavioral characteristics were then descriptively summarized for AGYW assigned to each group.

To examine the relationship between group membership and STI prevalence, a composite outcome variable–including prevalent HIV, syphilis, or chlamydia infection–was created given the limited number of infections reported in the overall sample. We fit a generalized linear model, specifying a binomial distribution and a log link to estimate prevalence, prevalence ratios (PR) and 95% confidence intervals (CI) for the relationship between each latent class group and the composite STI outcome. A minimally sufficient set of potential confounders were identified using a directed acyclic graph [48]; ultimately, age was identified as the only covariate for inclusion in the model. AGYW with missing or inconclusive HIV, syphilis, or chlamydia results were excluded (n = 15, 1.3%).

Analyses were conducted among the full sample of AGYW (N = 1,510). In sensitivity analyses, we repeated all analyses, including both the LCA and the subsequent outcome model, among only those AGYW who reported they were sexually active (n = 962).

## Results

### Description of AGYW

A total of 3,265 individuals were approached by the study team in Ethiopia from February 2018 to June 2019; 64 declined initial engagement with the study team and 57 had a language barrier, resulting in 3,144 individuals screened. Among those screened, 2,313 were eligible for study participation. Among those eligible, 812 (35.1% refused to participate) and 1,501 (64.9%) AGYW consented to study participation and were enrolled. Participants were split across Addis Ababa (n = 800, 53%) and Gambella Town (n = 701, 47%). The mean age at enrollment was 19.9 years (sd ±2.5); 956 (64%) were out-of-school, and 962 (64%) reported a history of sexual activity (Table 1). Almost two-thirds (n = 934, 62%) migrated into the city, of which, 51% reported migrating from a rural area. Just under one-quarter of all AGYW reported some food insecurity (n = 340, 23%), more than half (63%) were employed, and more than a quarter (25%) reported a history of physical violence. Relative frequencies for social- and structural-level factors–including schooling status, migration history, employment, orphan status, and food insecurity–were largely similar across AGYW irrespective of sexually active status.

**Table 1:** Characteristics of 1,501 adolescent girls and young women (AGYW) aged 15–24 years in Addis Ababa and Gambella Town, Ethiopia, 2018–2019.

	Total (N = 1,501)		Never had sex (N = 539)		Sexually active (N = 962)	
	n	%	n	%	n	%
Age						
15–19 years	689	46.0%	377	70.2%	312	32.5%
20–24 years	809	54.0%	160	29.8%	649	67.5%
Ever attended school	1,433	95.5%	519	96.3%	914	95.0%
Currently enrolled in school	545	36.4%	289	53.8%	254	26.7%
Migrated to current location	934	62.3%	309	57.3%	625	65.0%
Formally or informally employed	945	63.0%	273	50.6%	672	69.9%
No living parents	93	6.2%	20	3.7%	73	7.6%
Food insecurity^a^	340	22.7%	77	14.3%	263	27.3%
History of physical violence	395	26.4%	101	18.8%	294	30.6%
History of sexual violence	145	9.7%	12	2.2%	133	13.8%
Alcohol consumption in the past 12 months	545	36.3%	101	18.8%	444	46.2%
Moderate to severe depression	138	9.2%	25	4.7%	113	11.8%
Married	245	16.3%	3	0.6%	242	25.2%
Use of any hormonal or long-acting method of contraception^b^	572	38.1%	5	0.9%	567	58.9%
Ever tested for HIV	1,019	68.0%	261	48.5%	758	78.9%
Ever tested for STI	137	9.3%	7	1.3%	130	13.5%
Age disparate sexual partner^c^	624	41.6%	-	-	624	64.9%
Vaginal sex in last 12 months	784	52.2%	-	-	784	81.5%
Never or inconsistent condom use (vaginal sex)^d^	652	83.3%	-	-	652	83.3%
Transactional sex	175	11.7%	-	-	175	18.2%

^a^ Defined as going to sleep hungry in past 4 weeks

^b^ Injectable, oral contraceptive pill, intrauterine device or implant

^c^ Defined as a male partner ≥5 years older than AGYW

^d^ Among the 784 sexually active participants reporting having sex in the prior 12 months

The mean age of AGYW who were sexually active was 20.7 years (sd ±2.3), compared to 18.5 years (sd ±2.2) among those who reported never having sex. Among AGYW who were sexually active (64%), the mean age of sexual debut was 16.8 years (sd ±2.5). Most (n = 784, 82%) sexually active AGYW reported having sex within the prior 12 months, of which half (50%) reported never using a condom and 34% reported inconsistent condom use. History of pregnancy was reported among 351 (37%) of sexually active AGYW. Almost a fifth (18%) of sexually active AGYW reported a history of transactional sex.

### Vulnerability groups as identified through LCA

A three-class model emerged as the most optimal model to summarize social- and structural-level factors associated with HIV/STI acquisition among AGYW. Fit statistics for the full model are presented in the supplemental materials (S1 Table). The conditional probabilities and latent class proportions for the variables included in this model varied across groups, and are presented in Table 2. We labeled these groups as “Highly vulnerable” (comprising an estimated 21% of AGYW); “Socially and economically stable, out-of-school, migrants" (comprising an estimated 42%); and “Socially and economically stable, in-school, never migrated” (an estimated 37% of AGYW).

**Table 2:** Latent class proportions and conditional probabilities for a 3-class model among 1,501 adolescent girls and young women (AGYW) in Ethiopia aged 15–24, 2018–2019.

	Highly vulnerable	Economically and socially stable, out-of-school, migrants	Stable, in-school, never migrated
	**Latent class proportions**		
	0.21	0.42	0.37
	**Conditional probabilities**		
Schooling status			
In-school	0.07	0.09	0.83
Out-of-school	0.93	0.91	0.17
Migration			
Migrated	0.71	0.80	0.37
Never migrated	0.29	0.20	0.63
Food insecurity			
None	0.38	0.88	0.87
Yes, rarely	0.40	0.05	0.11
Yes, sometimes/often	0.22	0.07	0.02
Orphan status			
Both parents living	0.46	0.74	0.72
One parent living	0.37	0.23	0.24
No parents living	0.17	0.03	0.04
Social support (tertiles)			
First (low)	0.66	0.41	0.09
Second (medium)	0.29	0.29	0.41
Third (high)	0.05	0.30	0.50
Employment			
Unemployed	0.19	0.03	0.85
Informally employed	0.71	0.45	0.08
Employed in public/private sector	0.10	0.52	0.07

Based on the maximum posterior probability assignment rule, 225 (17%) AGYW were assigned to the highly vulnerable group, 692 (46%) to the stable, out-of-school, migrant group, and 554 (37%) to the stable, in-school, never migrated group (Table 3). These percentages approximated the group sizes estimated from the model’s parameters (as previously referenced in Table 2). For all groups, the median posterior probability of group membership was >0.80.

**Table 3:** Demographic, behavioral, and psychosocial characteristics by latent class analysis among 1,501 adolescent girls and young women aged 15–24 years in Ethiopia, 2018–2019^a^.

	Highly vulnerable N = 255 (17.0%)		Stable, out-of-school, migrants N = 692 (46.1%)		Stable, in-school, never migated N = 554 (36.9%)	
	Median	IQR	Median	IQR	Median	IQR
Posterior probability of latent class membership	0.82	0.68, 0.91	0.82	0.73, 0.96	0.99	0.99, 1.00
Age (continuous)	20	18, 23	20	18, 22	19	18, 21
	**n**	**%**	**n**	**%**	**n**	**%**
Age (categorical)						
15–19	95	37.3	281	40.7	314	56.8
20–24	160	62.8	410	59.3	239	43.2
Marital status						
Not married	217	85.1	580	84.1	457	82.5
Married	38	14.9	110	15.9	97	17.5
Depression						
Mild or none	203	79.6	641	92.8	517	93.5
Moderate or severe	52	20.4	50	7.2	36	6.5
Sexual debut^b^						
< 16 years	89	42.2	122	26.1	53	18.7
16–18 years	87	41.2	223	47.7	167	59.0
>18 years	35	16.6	123	26.3	63	22.3
Transactional sex						
No	166	65.1	611	88.4	548	98.9
Yes	89	34.9	80	11.6	6	1.1
Partner age difference^b^						
<5 years	53	25.1	159	34.0	126	44.5
5–10 years	83	39.3	226	48.3	136	48.1
>10 years	75	35.6	83	17.7	21	7.4
Physical or sexual violence						
No	125	49.0	480	69.6	429	77.6
Yes	130	51.0	210	30.4	124	22.4
Condom frequency^b^						
Inconsistent	135	64.0	311	66.3	206	72.8
Consistent	46	21.8	67	14.3	18	6.4
No sex, last 12 months	30	14.2	91	19.4	59	20.9
Prior pregnancy						
No	156	61.2	541	78.3	450	81.2
Yes	99	38.8	150	21.7	104	18.8

^a^ Missing: age 1; schooling status 11; marital status 2; orphan status 2; migration 2; depression 2; employment 5; transactional sex 1; physical or sexual violence 3; prior pregnancy 1

^b^ Among sexually active, n = 962

AGYW within the three groups differed with respect to sociodemographic and behavioral characteristics, including sexual risk behaviors (Table 3). Among AGYW in the highly vulnerable group, 42% reported first having sex while they were less than 16 years of age. More than one-third (34%) had engaged in transactional sex and 35% had engaged in sex with a partner that was >10 years older. Physical or sexual violence was common (51%), and 39% had previously been pregnant. Sexual risk behaviors were less prevalent and largely similar among AGYW in the other and more “stable” groups, though AGYW in the stable, in-school, non-migrant group were younger than the stable, out-of-school, migrant AGYW.

### Vulnerability profiles and STI prevalence

A total of 151 (10.2%) AGYW were diagnosed with one or more STIs at enrollment; 40 were living with HIV, 36 tested positive for syphilis, and 81 tested positive for chlamydia. STI prevalence ranged from 7.7% (95% CI 5.5, 9.9) to 19.0% (95% 14.7, 24.5) across groups. STI prevalence was generally similar among AGYW in each of the more “stable” groups (Table 4). AGYW who were classified as highly vulnerable had nearly twice the STI prevalence as AGYW who were stable, in-school, and had no reported history of migration (aPR 1.9; 95% CI 1.3, 2.8) (Table 4).

**Table 4:** Unadjusted and adjusted prevalence ratios (PR) and 95% confidence intervals (CI) for the association between vulnerability profiles and sexually transmitted infection (STI) among 1,501 adolescent girls and young women (AGYW) aged 15–24 in Ethiopia, 2018–2019^a^.

	No. infected^b^	Prevalence (95% CI)	PR (95% CI)	aPR (95% CI)^c^
Stable, in-school, never migrated	51	9.2 (7.1, 12.0)	1.	1.
Stable, out-of-school, migrated	52	7.7 (5.9, 9.9)	0.83 (0.57, 1.20)	0.78 (0.54, 1.13)
Highly vulnerable	48	19.0 (14.7, 24.5)	2.06 (1.43, 2.96)	1.93 (1.33, 2.80)

Abbreviations. No.: number, CI: confidence interval, PR: prevalence ratio, aPR: adjusted prevalence ratio

^a^ 15 AGYW had incomplete or missing HIV, syphilis, or chlamydia results and were excluded from effect estimates

^b^ Comprises a positive result for either HIV, syphilis, or chlamydia

^c^ Adjusted only for age, as identified using a directed acyclic graph

## Discussion

Distinct patterns of vulnerability, comprising higher order social and structural factors known to facilitate HIV/STI acquisition and transmission, were identified in this study of AGYW aged 15–24 in Ethiopia using LCA. We identified a distinctly and highly vulnerable group of AGYW, characterized by extreme social and structural vulnerability with respect to schooling status, migration history, food insecurity, orphanhood, social support, and employment. AGYW in this group had nearly twice the HIV/STI prevalence as other AGYW. These findings highlight the importance of recognizing additional social and structural factors, above and beyond sexual risk, in the development of targeted HIV/STI prevention programs and interventions for AGYW.

Other studies have demonstrated the role of schooling status, employment, migration, and social support in predicting risk and vulnerability to STIs in AGYW [22, 23, 25, 26, 43, 49–51]. A critical advancement of the present work is the exploration of patterns of how these factors present together to impact HIV/STI acquisition, irrespective of more proximal sociodemographic and behavioral determinants. In this analysis, AGYW who were identified as highly vulnerable had nearly twice the prevalence of HIV/STIs as AGYW who were socially more stable, even in the absence of a dedicated sexual-risk assessment. While sexual-risk behaviors such as condomless sex and sex with multiple partners can increase HIV/STI acquisition risks [50, 52], sexual decision-making is not siloed outside of the broader context of young women’s lives. Sexual behaviors exist amidst a complex fabric of other structural and individual factors that can potentiate vulnerability [51, 53, 54]. Moreover, once an AGYW tests positive for HIV or an STI, treatment programs should consider similar structural influences and risk factors which will also affect treatment initiation, retention, and adherence [53–58]. As such, HIV-prevention interventions for AGYW need not focus exclusively on sexual behavior, but instead should take a more holistic approach. Interventions that provide AGYW the economic and social support needed to meet their most basic needs may be most beneficial in supporting AGYW to make their own sexual and relationship choices, particularly before HIV exposure through transactional sex or intergenerational sex can occur.

Identifying classes of structural risk which map onto sexual and HIV acquisition risk is important for two key reasons. First, given social pressures to misreport sexual behavior, utilizing measures which are less culturally sensitize may improve measurement of exposure, while still linking to the overall health outcome (HIV/STIs). This can allow for easier identification of risks for poorer health outcomes upon which to intervene. Secondly, close review of the classes of risk reveal potential pathways through which young women may transition across the continuum of risk, potentially allowing for the identification of factors which lead to increased sexual risk at an earlier stage, again allowing for earlier intervention. For example, young women in the stable, in-school, non-migrant class appear in many ways similar to those in the stable, out-of-school, migrant class; however those in the former (in-school, non-migrant) class are younger and have sexual and violence risks comparable to that of the older, migrant out-of-school group, suggesting potentially that their paths may be at a crossroads and that intervention at this stage in which they are more readily reached may offer long-term benefits before social support declines and migration and transactional sex increase. Interventions may vary, but have included economic and/or women’s empowerment, violence prevention, focus on building strong communication habits, or holistic adolescent sexual and reproductive health services which are sex-positive and focus on adolescent development [59–61].

Building on work demonstrating the influence of transactional sex on HIV acquisition risks [15, 18, 62], these analyses also highlight the distinct structural- and individual-level patterns of risk among AGYW engaged in transactional sex. Intervening earlier on the spectrum of risk–including the employment of differentiated program science approaches and a full range of prevention options–is relevant to prevent HIV acquisition and to provide younger women in socially and economically volatile situations additional support options before their pathways lead to increased risks [11, 63, 64]. Social support, parental loss, food insecurity and migration were critical structural factors associated with this high vulnerability group; programs to intervene with adolescents and their families prior to migration to confer skills to avoid violence and create safe economic options may be important, alongside programs for those who have migrated to address food insecurity and create social support and positive guidance in the absence of parental involvement [65].

Potential limitations of our study include the inability to infer causality between the classes of risk and the HIV/STI outcome given the utilization of cross-sectional data, social desirability and reporting bias, and the inability to examine the relationship between vulnerability profiles and HIV prevalence independently given the limited number of infections recorded in our data. Social desirability and reporting bias may have led to a potential misclassification of sexual history. For example, STI prevalence among those reporting no history of sexual activity was comparable to that of those reported to be sexually active. However, this further highlights the importance of identifying other less stigmatized items which may be less affected by social desirability bias, and which may provide similar insight into HIV and STI risk. Moreover, evening hours for recruitment in Gambella Town were limited due to safety considerations, potentially affecting the sample of young women sampled in that region. Even so, findings were comparable across areas and HIV/STI prevalence were in fact higher in Gambella, suggesting that at-risk AGYW were reached in both locations. Individuals within venues may be more similar in their characteristics and risks as compared to AGYW at other venues; while venues were not accounted for in this analysis, previously published results from this study found that vulnerability was high across venues [38]. Finally, results of this analysis may not be generalizable outside of the two cities sampled or beyond Ethiopia. Nevertheless, given the strong associations between STIs and HIV and the comparable pathways of risk across settings [66–68], the results remain relevant within the Ethiopian context and the approach for identifying patterns of vulnerability and classes of structural risks among AGYW can be replicated in other settings.

## Conclusion

As efforts to achieve HIV epidemic control continue, leveraging a nuanced understanding of HIV risk heterogeneity among AGYW to tailor HIV programming responses will be increasingly important in Ethiopia and in countries across sub-Saharan Africa. Moreover, without intensified and innovative efforts to address the burden of HIV among youth, there is no possibility for ending the HIV epidemic. A strength of this analysis is that it allows for a more holistic conceptualization of vulnerability, including upstream structural and social factors that may drive risk of HIV and other STIs. Assessments of vulnerability that extend beyond measures of sexual risk may hold promise for altering trajectories of risk earlier on in a young woman’s life cycle. From an intervention-development perspective, early identification of AGYW who may be at highest risk of HIV and STI acquisition and transmission, even in the absence of reported sexual activity, is essential for achieving epidemic control.

## Supporting information

S1 Table Fit statistics comparing 2–6 class latent class models of social and structural determinants of HIV/STI acquisition among 1,501 adolescent girls and young women (AGYW) in Ethiopia aged 15–24, 2018–2019. (DOCX)

S2 Table Fit statistics comparing 2–6 class latent class models of social and structural determinants of HIV/STI acquisition among 962 sexually active adolescent girls and young women (AGYW) in Ethiopia aged 15–24, 2018–2019. (DOCX)

S3 Table Latent class proportions and conditional probabilities for a 3-class model among 962 sexually active adolescent girls and young women (AGYW) in Ethiopia aged 15–24, 2018–2019. (DOCX)

S4 Table Demographic, behavioral, and psychosocial characteristics by latent class analysis group among 962 sexually active adolescent girls and young women aged 15–24 years in Ethiopia, 2018–2019. (DOCX)

S5 Table Unadjusted and adjusted prevalence ratios (PR) and 95% confidence intervals (CI) for the association between vulnerability profiles and sexually transmitted infection (STI) among 962 sexually active adolescent girls and young women (AGYW) aged 15–24 in Ethiopia, 2018–2019. (DOCX)
