# Correction: Vulnerability profiles and prevalence of HIV and other sexually transmitted infections among adolescent girls and young women in Ethiopia: A latent class analysis

**DOI:** 10.1371/journal.pone.0236910

**Published:** 2020-07-23

**Authors:** Carly A. Comins, Katherine B. Rucinski, Stefan Baral, Samuele A. Abebe, Andargachew Mulu, Sheree R. Schwartz

In the Funding section, the grant number from the funder Project SOAR is listed incorrectly. The correct grant number is: Cooperative agreement AID-OAA-A-14-00060.
