# Supplementary material for: Vulnerability profiles and prevalence of HIV and other sexually transmitted infections among adolescent girls and young women in Ethiopia: A latent class analysis
Source: PLoS One. 2020 May 14;15(5):e0232598. doi: 10.1371/journal.pone.0232598 (PMC7224533; doi:10.1371/journal.pone.0232598)
Supplement: S1 Table — (DOCX) [file pone.0232598.s001.docx]

**S1 Table.** **Fit statistics comparing 2-6 class latent class models of structural determinants of HIV acqusition among 1,501 adolescent girls and young women (AGYW) in Ethiopia aged 15-24, 2018-2019**

| **Classes** | **DF** | **G^2^** | **AIC** | **BIC** | **Entropy** | **Percentage of seeds associated with best fitting model** |
| --- | --- | --- | --- | --- | --- | --- |
| 1 | - | - | - | - | - | - |
| 2 | 302 | 492.34 | 534.34 | 645.93 | 0.77 | 100.00 |
| 3 | 291 | 373.46 | 437.46 | 607.50 | 0.66 | 100.00 |
| 4 | 280 | 322.51 | 408.51 | 637.01 | 0.64 | 56.00 |
| 5 | 269 | 291.16 | 399.16 | 686.11 | 0.62 | 17.00 |
| 6 | 258 | 271.06 | 401.06 | 746.46 | 0.67 | 48.00 |
