# Supplementary material for: Vulnerability profiles and prevalence of HIV and other sexually transmitted infections among adolescent girls and young women in Ethiopia: A latent class analysis
Source: PLoS One. 2020 May 14;15(5):e0232598. doi: 10.1371/journal.pone.0232598 (PMC7224533; doi:10.1371/journal.pone.0232598)
Supplement: S2 Table — (DOCX) [file pone.0232598.s002.docx]

**S2 Table.** Fit statistics comparing 2-6 class latent class models of social and structural determinants of HIV/STI acquisition among 962 sexually active adolescent girls and young women (AGYW) in Ethiopia aged 15-24, 2018-2019

| **Classes** | **DF** | **G^2^** | **AIC** | **BIC** | **Entropy** | **Percentage of seeds associated with best fitting model** |
| --- | --- | --- | --- | --- | --- | --- |
| 1 | - | - | - | - | - | - |
| 2 | 302 | 381.63 | 423.63 | 525.88 | 0.71 | 100.00 |
| 3 | 291 | 292.16 | 356.16 | 511.97 | 0.63 | 63.00 |
| 4 | 280 | 262.01 | 348.01 | 557.38 | 0.58 | 58.00 |
| 5 | 269 | 244.14 | 352.14 | 615.06 | 0.63 | 67.00 |
| 6 | 258 | 225.37 | 355.37 | 671.85 | 0.63 | 68.00 |
