# Supplementary material for: Vulnerability profiles and prevalence of HIV and other sexually transmitted infections among adolescent girls and young women in Ethiopia: A latent class analysis
Source: PLoS One. 2020 May 14;15(5):e0232598. doi: 10.1371/journal.pone.0232598 (PMC7224533; doi:10.1371/journal.pone.0232598)
Supplement: S3 Table — (DOCX) [file pone.0232598.s003.docx]

**S3 Table.** Latent class proportions and conditional probabilities for a 3-class model among 962 sexually active adolescent girls and young women (AGYW) in Ethiopia aged 15-24, 2018-2019

|  | **Highly vulnerable** | **Stable, out-of-school, migrants** | **Stable, in school, never migrated** |
| --- | --- | --- | --- |
|  | **Latent class proportions** | | |
|  | 0.22 | 0.43 | 0.35 |
|  | **Conditional probabilities** | | |
| Schooling status |  |  |  |
| in-school | 0.02 | 0.06 | 0.67 |
| out-of-school | 0.98 | 0.94 | 0.33 |
| Migration |  |  |  |
| migrated | 0.69 | 0.85 | 0.39 |
| never migrated | 0.31 | 0.15 | 0.61 |
| Food insecurity |  |  |  |
| none | 0.36 | 0.81 | 0.86 |
| Yes, rarely | 0.44 | 0.08 | 0.12 |
| Yes, sometimes or often | 0.21 | 0.12 | 0.03 |
| Orphan status |  |  |  |
| both parents living | 0.39 | 0.71 | 0.62 |
| one parent living | 0.41 | 0.26 | 0.33 |
| no parents living | 0.20 | 0.03 | 0.05 |
| Social support (tertiles) |  |  |  |
| first (low) | 0.68 | 0.47 | 0.14 |
| second (medium) | 0.27 | 0.27 | 0.46 |
| third (high) | 0.05 | 0.26 | 0.40 |
| Employment |  |  |  |
| unemployed | 0.18 | 0.01 | 0.74 |
| informally employed | 0.78 | 0.48 | 0.15 |
| Employed in public/private sector | 0.05 | 0.51 | 0.11 |
