# Supplementary material for: Vulnerability profiles and prevalence of HIV and other sexually transmitted infections among adolescent girls and young women in Ethiopia: A latent class analysis
Source: PLoS One. 2020 May 14;15(5):e0232598. doi: 10.1371/journal.pone.0232598 (PMC7224533; doi:10.1371/journal.pone.0232598)
Supplement: S4 Table — (DOCX) [file pone.0232598.s004.docx]

**S4 Table.** Demographic, behavioral, and psychosocial characteristics by latent class analysis group among 962 sexually active adolescent girls and young women aged 15-24 years in Ethiopia, 2018-2019^a,b^

|  | **Highly vulnerable**  **N=186 (19.3%)** | | **Stable, out-of-school, migrants**  **N=453 (47.1%)** | | **Stable, in school, never migrated**  **N=323 (33.3%)** | |
| --- | --- | --- | --- | --- | --- | --- |
|  | **Median** | **IQR** | **Median** | **IQR** | **Median** | **IQR** |
| Posterior probability  of latent class membership | 0.75 | 0.66, 0.90 | 0.81 | 0.69, 0.95 | 0.98 | 0.91, 1.00 |
| Age (continuous) | 21 | 19, 23 | 21 | 19, 23 | 20 | 19, 22 |
|  | **n** | **%** | **n** | **%** | **n** | **%** |
| Age (categorical) |  |  |  |  |  |  |
| 15-19 | 59 | 31.7 | 111 | 34.4 | 142 | 31.4 |
| 20-24 | 127 | 68.3 | 212 | 65.6 | 310 | 68.6 |
| Marital status |  |  |  |  |  |  |
| not married | 152 | 81.7 | 215 | 66.6 | 352 | 77.9 |
| married | 34 | 18.3 | 108 | 33.4 | 100 | 22.1 |
| Depression |  |  |  |  |  |  |
| mild or none | 147 | 79.0 | 296 | 91.9 | 405 | 89.4 |
| moderate or severe | 39 | 21.0 | 26 | 8.1 | 48 | 10.6 |
| Sexual debut |  |  |  |  |  |  |
| < 16 years | 78 | 41.9 | 62 | 19.2 | 124 | 37.4 |
| 16-18 years | 80 | 43.0 | 182 | 56.4 | 215 | 47.5 |
| >18 years | 28 | 15.1 | 79 | 24.5 | 114 | 25.2 |
| Transactional sex |  |  |  |  |  |  |
| no | 108 | 58.1 | 313 | 96.9 | 366 | 80.8 |
| yes | 78 | 41.9 | 10 | 3.1 | 87 | 19.2 |
| Partner age difference |  |  |  |  |  |  |
| <5 years | 48 | 25.8 | 139 | 43.0 | 151 | 33.3 |
| 5-10 years | 73 | 39.3 | 160 | 49.5 | 212 | 46.8 |
| >10 years | 65 | 35.0 | 24 | 7.4 | 90 | 19.9 |
| Physical or sexual violence |  |  |  |  |  |  |
| no | 87 | 46.8 | 242 | 75.2 | 274 | 60.6 |
| yes | 99 | 53.2 | 80 | 24.8 | 178 | 39.4 |
| Condom frequency |  |  |  |  |  |  |
| inconsistent | 118 | 63.4 | 237 | 73.4 | 297 | 65.6 |
| consistent | 41 | 22.0 | 20 | 6.2 | 70 | 15.5 |
| no sex, last 12 months | 27 | 14.5 | 66 | 20.4 | 86 | 19.0 |
| Prior pregnancy |  |  |  |  |  |  |
| no | 98 | 52.7 | 207 | 64.1 | 306 | 68.0 |
| yes | 88 | 47.3 | 116 | 35.9 | 147 | 32.5 |

^a^ Missing: age 1; marital status 1; depression 1; physical or sexual violence 1

^b^ Each AGYW was assigned to the group for which her posterior probability of group membership was highest.
