# Supplementary material for: Vulnerability profiles and prevalence of HIV and other sexually transmitted infections among adolescent girls and young women in Ethiopia: A latent class analysis
Source: PLoS One. 2020 May 14;15(5):e0232598. doi: 10.1371/journal.pone.0232598 (PMC7224533; doi:10.1371/journal.pone.0232598)
Supplement: S5 Table — (DOCX) [file pone.0232598.s005.docx]

**S5 Table.** Unadjusted and adjusted prevalence ratios (PR) and 95% confidence intervals (CI) for the association between vulnerability profiles and sexually transmitted infection (STI) among 962 sexually active adolescent girls and young women (AGYW) aged 15-24 in Ethiopia, 2018-2019

|  | **No. infected^a^** | **Prevalence (95% CI)** | **PR (95% CI)** | **aPR (95% CI)^b^** |
| --- | --- | --- | --- | --- |
| Stable, in school,  never migrated | 29 | 9.0 (6.4, 12.7) | 1. | 1. |
| Stable, out-of-school, migrants | 42 | 9.5 (7.1, 12.6) | 1.05 (0.67, 1.65) | 1.05 (0.67, 1.65) |
| Highly vulnerable | 41 | 22.3 (17.0, 29.2) | 2.47 (1.59, 3.84) | 2.47 (1.60, 3.85) |

Abbreviations. No.: number, CI: confidence interval, PR: prevalence ratio, aPR: adjusted prevalence ratio

^a^ Comprises a positive result for either HIV, syphilis, or chlamydia

^b^ Adjusted only for age, as identified using a directed acyclic graph
